# Magnetic Stress Monitoring Using a Directional Potential Drop Technique

**DOI:** 10.1007/s10921-018-0510-4

**Published:** 2018-07-20

**Authors:** J. Corcoran, P. B. Nagy

**Affiliations:** 1 Department of Mechanical Engineering, Imperial College London, London, SW7 2AZ UK; 2 Department of Aerospace Engineering and Engineering Mechanics, University of Cincinnati, Cincinnati, OH 45221-0070 USA

**Keywords:** Potential drop, DCPD, ACPD, Magnetic permeability, Structural health monitoring, Stress measurement, Strain measurement

## Abstract

An alternating current potential drop technique is presented that exploits anisotropic magnetostriction to monitor changes in applied stress in steel. The background to the technique is provided together with an ad hoc approximation that describes the sensitivity of the sensor to the underlying properties. A uniaxial loading experiment has been conducted on duplex and mild steel specimens showing that changes in stress are measureable. Saturation and hysteresis afflict the measurement, which, in addition to sensitivity to temperature and magnetisation, may undermine inversion. With the capabilities and limitations of the proposed technique introduced, guidance on possible suitable applications are given, concluding that use for monitoring the number and relative size of fatigue stress cycles may be a suitable and attractive opportunity.

## Introduction

Monitoring stress is potentially of great value to structural integrity engineers. Static stresses may drive plastic deformation, creep damage [12] or creep crack growth [28], while dynamic stresses may lead to fatigue [6]. It is common to infer stresses through measurement of elastic strain. By far the most prevalent means of monitoring stresses is the bondable foil strain gauge [15], yet they are unsuitable for many industrial applications where good adhesion to the surface is not possible, or in harsh environments. Advances in the field of in situ stress monitoring are therefore worthy of exploration.

The strain dependence of magnetic permeability has been exploited for stress measurements in the past; in this study it will be assumed that stress may be inferred from elastic strain according to Hooke’s Law. Brennan & Dover et al. developed an alternating current field measurement (ACFM) technique based on this principle [7, 14, 31]. A similar ACFM technique was also developed for the purpose of residual stress measurement in rails [5]. In the prior studies, it is noted that the permeability changes that arise from the application of elastic strain are strongly anisotropic, and it is anisotropy that will be utilised for inferring stress.

Potential drop measurements are a standard means of monitoring the geometric changes associated with the progress of damage in metallic engineering components, particularly crack growth [26] but also gross deformation such as creep strain [10]. Potential drop techniques lend themselves to permanent monitoring in harsh environments due to the simple and robust hardware required at component level. Using potential drop measurements to also infer stress is attractive as it may be obtained either as a by-product of an existing potential drop measurement or as a dedicated sensor for when strain gauges are infeasible.

Alternating current (AC) measurements are subject to the skin effect. The current is electromagnetically constricted to the surface of the component, resulting in an exponentially decreasing current density with depth. In the simple homogeneous isotropic case, the skin-depth, $\delta$, the depth at which the current density is $1/e$ (~ 37%) of the surface density, for the case of a half-space, is given by,

1 $$ \delta = \frac{1}{{\sqrt {\sigma \pi f\mu } }} $$

where $\sigma$ is the electrical conduictivity, $f$ is the alternating current frequency and $\mu$ is the magnetic permeability of the component. In non-magnetic materials the skin-effect may be utilised to concentrate the current nearer the surface for improved sensitivity to surface defects. In ferromagnetic materials, the magnetic permeability is highly variable so direct current (DC) or quasi-DC measurements are usually used in preference to AC due to the enhanced stability. One of the variables that the magnetic permeability is sensitive to is elastic strain through the ‘inverse magnetostrictive effect’; it is proposed in this paper that this sensitivity is exploited so that information on applied stress is obtained from AC potential drop measurements. The potential drop technique proposed uses permanently attached electrodes which are spot-welded to the component; this effectively uses the material ‘as the sensor’, and negates spatial material variation that may otherwise introduce unacceptable levels of uncertainty.

Unfortunately, the magnetic permeability in ferromagnetic materials is also known to be sensitive to many other variables, most notably temperature and magnetisation, as well as microstructural material parameters. Specificity is therefore potentially an issue that will need to be addressed in assessing the viability of magnetic based stress measurements.

In this paper the background to multi-frequency four-point potential drop measurements will be given to introduce the proposed technique; this will be followed by an ad hoc approximation that will be rigorously validated by computational simulation relating anisotropic permeability and conductivity to potential drop impedance measurements. A uniaxial loading experiment will be presented showing the stress sensitivity of the technique, followed by a demonstration of the sensitivity to temperature and magnetisation in an attempt to establish whether the technique is likely to have sufficient selectivity for practical applications.

## Multi-Frequency Four-Point Potential Drop Measurements

**Fig. 1:**
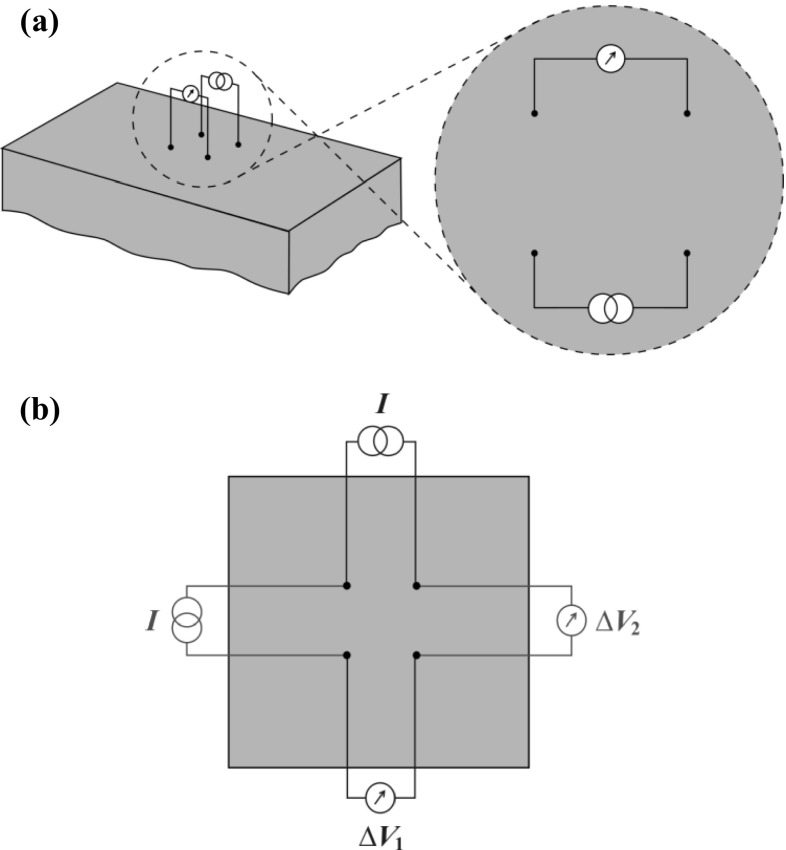
Illustration showing the square electrode configuration potential drop measurement. **a** shows a three-dimensional illustration of a four point potential drop measurement, **b** shows that two orthogonal potential drop measurements may be taken sequentially using the same four electrodes

Potential drop measurements require four electrodes that are galvanically connected to the surface of the test component. A known electrical current, $I$, is injected between two of the electrodes while the resulting electrical potential drop, $\Delta V$, is measured across the remaining two electrodes. The impedance, $Z$, can then be calculated,

2 $$ Z = \frac{\Delta V}{I} = R + iX $$

where $R$ is the resistance and $X$ is reactance by definition. Figure 1a shows one possible configuration of the electrodes; the electrodes form a square with current injecting electrodes forming one side of the square and the potential sensing electrodes forming the opposite side. This arrangement is known to have superior sensitivity to anisotropic changes in electrical conductivity than the more conventional ‘in-line’ arrangement where the electrodes are all arranged along a straight line [24]; the following section will show that the square configuration of electrodes is also sensitive to anisotropic changes in magnetic permeability. A further useful feature of the square configuration of electrodes is that two orthogonal measurements can be taken sequentially at a given location using just four electrodes, as illustrated in Fig. 1b. The enhanced directionality, combined with the ability to take orthogonal measurements permits the detection of stress-dependent changes in anisotropic material properties, including magnetic permeability, which will be central to this study.

**Fig. 2:**
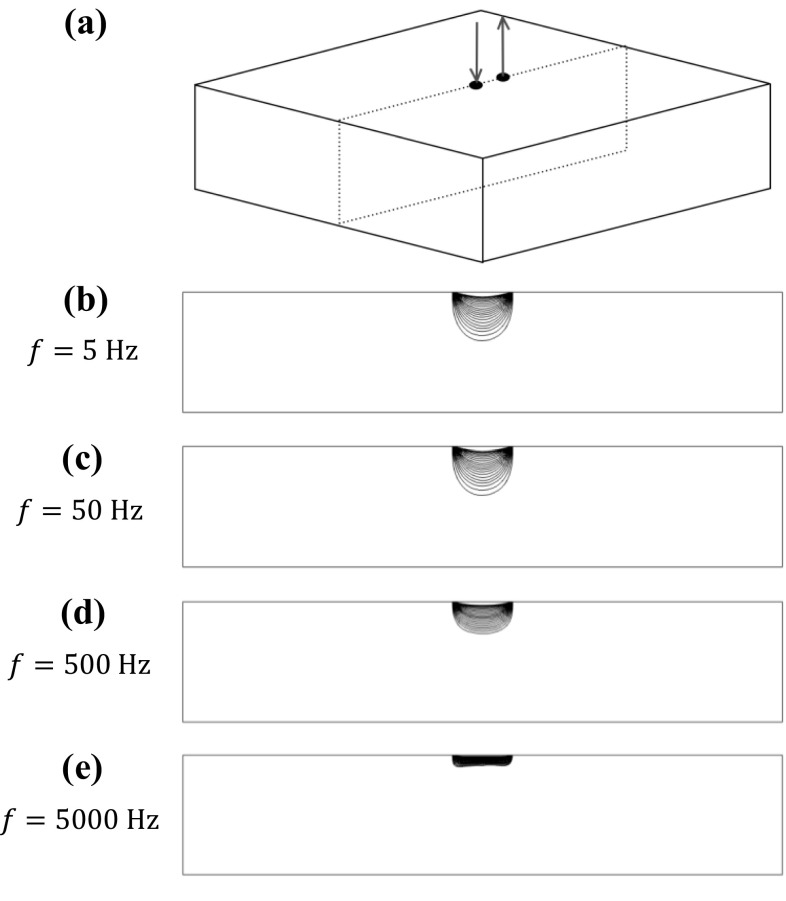
Finite element simulation results obtained using finite element simulations. **a** Current is injected between two points separated by 10 mm on the surface of a component with dimensions of 100 mm x 100 mm x 20 mm, with relative magnetic permeability of 1 and conductivity of 100%IACS. **b**–**e** Streamlines show the current path for a range of frequencies

The frequency dependence of potential drop measurements in electromagnetically isotropic materials has been previously reported [3, 11], while the frequency dependence of potential drop measurements in anisotropic materials is developed in this paper. Figure 2 shows the result of a Comsol Comsol [9] finite element simulation intended to illustrate the frequency dependence of current distribution in an isotropic material. Current of increasing frequency is injected between two points on the surface of a cuboid. The current distributions are shown illustrating the behaviour is split into two regimes, quasi-DC and AC. At high, AC, frequencies the current penetration is limited by the skin effect according to Eq. (1). As the frequency is decreased to quasi-DC frequencies the skin depth will become large and the current distribution will tend to the DC distribution where the penetration is limited not by the skin effect but by geometry. In the example of Fig. 2 the current is confined largely within a depth of half the electrode separation.

**Fig. 3:**
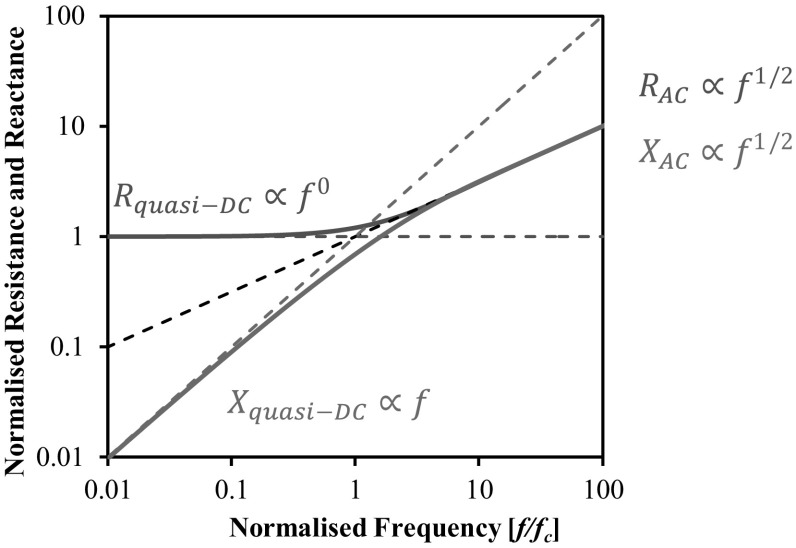
Frequency response of impedance for a square electrode configuration potential drop measurement, as illustrated in Fig. 1. Resistance and reactance are normalised by the DC resistance. Frequency is normalised to the corner frequency which is approximately equal to the transition frequency [11]. The analytical solution is based on that by Bowler [2] and is presented in [11]

Figure 3 shows the frequency dependence of a square electrode configuration potential drop measurement. The frequency at which the skin depth equals the current limiting geometric dimension will be referred to as the transition frequency. The transition frequency divides the AC and DC-like behaviour with a transitional regime. At ‘quasi-DC’ frequencies, below the transition frequency, the current penetration will no longer be dictated by the skin effect and therefore resistance measurements will only be sensitive to electrical resistivity and the geometry changes associated with material damage. Quasi-DC resistance measurements are therefore effectively independent of frequency as shown in Fig. 3. Above the transition frequency the current penetration is dictated by the skin effect and therefore additionally becomes sensitive to changes in stress-dependent magnetic permeability; the electrical resistance and reactance are proportional to the square root of frequency as shown. It is proposed in this study that quasi-DC measurements, holding information on resistivity and geometry, may be combined with AC measurements, holding information on magnetic permeability in addition to resistivity and geometry to provide a stress-dependent measurement.

It is believed that the key to monitoring stress-dependent magnetic permeability changes is the anisotropy, i.e. stress-induced directional changes in magnetic permeability. The directional changes in magnetic permeability require a more sophisticated description of the skin depth than the isotropic description provided in Eq. (1); the directional changes in permeability will influence the lateral spread of current in addition to the depth.

**Fig. 4:**
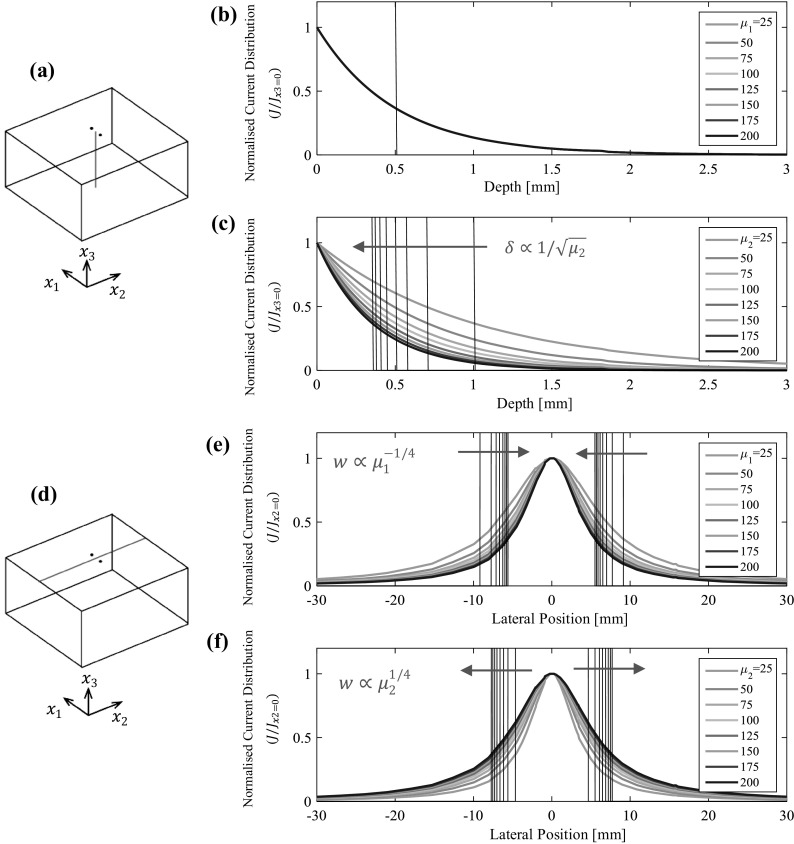
Finite element simulations of the current density resulting from anisotropic changes in magnetic permeability; the relative magnetic permeability in all directions not indicated in the legend is 100. Two point current source electrodes are separated by 10 mm on a 50 mm thick block of material of conductivity 5.07 × 10^6^ S/m (8.74%IACS). **a** and **d** illustration of the model geometry. **b** and **c** Current distribution through the depth of the component, with permeability changes parallel and normal to electrode alignment respectively. **e** and **f** Current distribution across the width of the component, with permeability changes parallel and normal to electrode alignment respectively

To illustrate the influence of anisotropic permeability changes on the current distribution of an AC potential drop measurement a finite element study was completed. Two point current source electrodes were modelled on a block of material, as illustrated in Fig. 4. The magnetic permeability was then anisotropically manipulated so that the magnetic permeability aligned in the direction of the electrodes was altered, followed by the magnetic permeability normal to the electrode alignment. The current distribution through the depth of the component along the bisector line between the injection points (see Fig. 4a) and along the top surface of the block normal to the electrode alignment (see Fig. 4d) is presented; the current density is normalised to the maximum current density at each permeability state.

Referring to the current distribution through the depth of the component (Fig. 4a–c), increases in permeability in either direction increases the current density at the surface, but Fig. 4b shows that the current *distribution* is unaffected by changes in magnetic permeability in the direction of electrode alignment; the ‘effective’ skin depth (the point at which the current density reduces to $1/e$ of the surface current density) is unchanged. Figure 4c shows that the skin depth is inversely proportional to the square root of the magnetic permeability in the direction normal to the electrode alignment, this is equivalent to the isotropic case of Eq. (1).

To aid analysis of the lateral current distribution (Fig. 4d–f) a new term is introduced, the ‘skin width’, $w$, which is analogous to the well-known ‘skin depth’, $\delta$, and is defined as the lateral distance from the point halfway between the two electrodes to the point where the current density is $1/e$ of the central current density. From the simulation results shown in Fig. 4e and f it is evident that the skin width is proportional to the fourth-root of the magnetic permeability normal to electrode alignment ($w \propto \mu_{2}^{1/4}$) and inversely proportional to the fourth-root of the magnetic permeability in the direction of electrode alignment ($w \propto \mu_{1}^{ - 1/4}$). The magnetic permeability normal to the surface plane (in the $x_{3}$ direction) has no effect on the current distribution; this directionality is expected since the magnetic field is always orthogonal to the current that produced it.

In order to calculate the impedance of an AC potential drop measurement the influence of both the skin width and depth would need to be combined. The following section provides an ad hoc approximation for square electrode configuration potential drop measurements of electromagnetically anisotropic materials.

## Ad Hoc Approximation of ACPD Measurements in Magnetically and Electrically Anisotropic Materials

Although exact solutions could be obtained following the formalism developed by Zhou and Dover [31], our goal here is to produce only a simplistic ad hoc approximation that captures the main features of the problem at hand. This approximation then will be validated numerically by comparison to finite element simulations.

### Low-Frequency ACPD in Thin Plates

#### Isotropic Plates

First, let us consider a thin plate of thickness,$t$, made of a material of homogeneous and isotropic magnetic permeability,$\mu$, and electric conductivity, $\sigma$. Alternating current, $I$, is injected at the origin of a cylindrical coordinate system ($r, \theta ,z$) on the bottom surface of the plate. The inspection frequency, $f$, is low enough so that the electromagnetic skin depth $\delta = 1/\sqrt {\pi f\mu \sigma }$ is much larger than the plate thickness, $t$, therefore quasi-static approximations can be used. Under such conditions, the current density beyond the immediate vicinity of the injection point is strictly radial and inversely proportional to the radial coordinate $r$ while it is uniform in the z and $\theta$ directions.

3 $$ J\left( r \right) = \frac{I}{2\pi rt} $$

The radial electrical field is

4 $$ E\left( r \right) = \frac{I\rho }{2\pi rt} $$

where we introduced the electric resistivity $\rho = 1/\sigma$. The resulting potential distribution is

5 $$ V\left( r \right) = \int \limits_{r}^{\infty } E\left( r \right)dr = \frac{I\rho }{2\pi t} \int \limits_{r}^{\infty } \frac{1}{r}dr = - \frac{I\rho }{2\pi t}\ln r + V_{0} $$

The transfer resistance can be determined by superposition as follows

6 $$ R = \frac{\Delta V}{I} = - \frac{\rho }{2\pi t}\left[ {\ln r_{11} - \ln r_{12} - \ln r_{21} + \ln r_{22} } \right] $$

where $r_{ij}$ denotes the distance from the $i$-th injection point to the $j$-th sensing point ($i$,  $j$  = 1, 2).

#### Anisotropic Plates

Tatarnikov [27] derived the electric potential distribution in a thin anisotropic plate as

7 $$ V\left( r \right) = - I\frac{{\sqrt {\rho_{1} \rho_{2} } }}{2\pi t}\ln \sqrt {\rho_{1}  x_{1}^{2} + \rho_{2}  x_{2}^{2} } + V_{0} $$

where $\rho_{1}$ and $\rho_{2}$ denote the electric resistivities in the $x_{1}$ and $x_{2}$ principal in-plane directions. When a square-electrode PD probe is aligned with the principal directions of the electric anisotropy in the plate, the transfer resistances $R_{1}$ and $R_{2}$ measured in the $x_{1}$ and $x_{2}$ directions are [21],

8a $$ R_{1} = \frac{{\sqrt {\rho_{1} \rho_{2} } }}{2\pi t}\ln \left( {1 + \frac{{\rho_{1} }}{{\rho_{2} }}} \right) $$

and

8b $$ R_{2} = \frac{{\sqrt {\rho_{1} \rho_{2} } }}{2\pi t}\ln \left( {1 + \frac{{\rho_{2} }}{{\rho_{1} }}} \right) $$

respectively.In the absence of stress-induced electrical anisotropy ($\rho_{1} = \rho_{2} = \rho$)

9 $$ R_{10} = R_{20} = \frac{\rho }{2\pi t}\ln \left( 2 \right) $$

Then, the normalized resistances can be obtained as follows

10a $$ \frac{{R_{1} }}{{R_{10} }} = \frac{{\sqrt {\rho_{1} \rho_{2} } }}{\rho }\frac{{\ln \left( {1 + \frac{{\rho_{1} }}{{\rho_{2} }}} \right)}}{{{ \ln }\left( 2 \right)}} $$

and

10b $$ \frac{{R_{2} }}{{R_{20} }} = \frac{{\sqrt {\rho_{1} \rho_{2} } }}{\rho }\frac{{\ln \left( {1 + \frac{{\rho_{2} }}{{\rho_{1} }}} \right)}}{\ln \left( 2 \right)} $$

The ratio of the two orthogonal resistances is then,

11 $$ \frac{{R_{1} }}{{R_{10} }}/ \frac{{R_{2} }}{{R_{20} }} = \frac{{\ln \left( {1 + \frac{{\rho_{1} }}{{\rho_{2} }}} \right)}}{{\ln \left( {1 + \frac{{\rho_{2} }}{{\rho_{1} }}} \right)}} $$

**Fig. 5:**
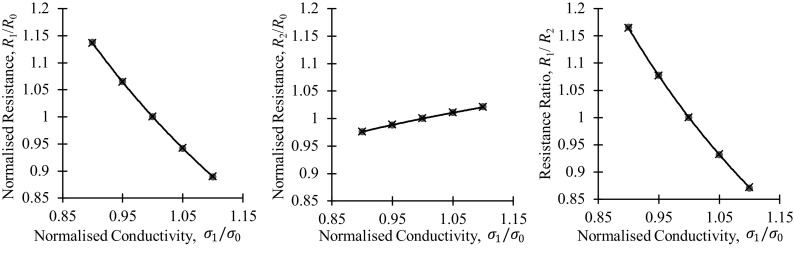
Comparison of simulated results (red circles) and the approximations of Eq. (10) and (11) (black lines and crosses) for a square electrode configuration DC potential drop measurement in an electrically anisotropic thin plate. The conductivity in the $\varvec{x}_{1}$ direction was varied by ± 10% while the conductivity in the other directions was kept constant (Color figure online)

This exact theoretical prediction is illustrated using finite element simulation as shown in Fig. 5; the agreement validates the model. The finite element model is of a square electrode configuration on an electrically anisotropic plate of thickness 2 mm and electrode separation 10 mm.

### High-Frequency ACPD in Thin Plates

#### Isotropic Plates

Next, let us assume that a high-frequency alternating current $I$ is injected into a thick plate at a sufficiently high frequency $f$ so that the electromagnetic skin depth is much smaller than the plate thickness ($\delta$ ≪ $t$), therefore thin-skin approximations can be used. The $r$-$\theta$ plane of the cylindrical coordinate system is chosen to coincide with the bottom surface of the thick plate that effectively occupies the $z > 0$ half-space. The distribution of the electric field in a conducting half-space due to alternating current injected at the surface has been analytically solved by Bowler [1, 2]. Based on her exact analytical solution of the electric current distribution, in the far-field of the injection point the conduction current density is purely radial

12 $$ J\left( {r,z} \right) = \left( {1 - i} \right)\frac{I}{2\pi r\delta }e^{ikz} $$

where the complex wave number $k$ is defined as

13 $$ k^{2} = i\omega \mu_{0} \sigma_{0} $$

Here, $k = \left( {1 + i} \right)/ \delta$, where $\delta$ is the previously defined electromagnetic skin depth and we continue to use the $\exp \left( { - i\omega t} \right)$ convention for the time dependence of harmonic temporal variation. Of course, at any distance $r$ from the injection electrode, the total integrated radial current is still equal to the injected current that is assumed to be positive when flowing into the half-space at the source

14 $$ 2\pi r\mathop \int \limits_{0}^{\infty } J_{r} \left( z \right)dz = I $$

The radial electric field is

15 $$ E\left( {r,z} \right) = \left( {1 - i} \right)I\frac{\rho }{2\pi r\delta }e^{ikz} $$

The resulting potential distribution in the far field of the injection point on the surface of the specimen ($$ z = 0) $$ is

16 $$ V\left( r \right) = - \left( {1 - i} \right)I\frac{\rho }{2\pi r\delta }\ln r + V_{0} $$

The real part of the complex transfer impedance can be determined by superposition as follows

17 $$ R = {\text{Re}}\left\{ {\frac{\Delta V}{I}} \right\} = - \frac{\rho }{2\pi \delta }\left[ {\ln r_{11} - \ln r_{12} - \ln r_{21} + \ln r_{22} } \right] $$

The close analogy between Eqs. (6) and (17) when the electromagnetic skin depth is substituted for the plate thickness ($t \to \delta$) has been previously exploited for deriving simple approximations for high-frequency ACPD in thick isotropic plates [11].

#### Anisotropic Plates

In this section we are going to further exploit the analogy between Eqs. (6) and (17) by extending the previous substitution for isotropic materials

18 $$ \frac{\rho }{t} \to \frac{\rho }{\delta } = \sqrt {\pi f\rho \mu } $$

to anisotropic materials in the following similar form

19a $$ \frac{{\rho_{1} }}{\delta } \to \sqrt {\pi f\rho_{1} \mu_{2} } $$

and

19b $$ \frac{{\rho_{2} }}{\delta } \to \sqrt {\pi f\rho_{2} \mu_{1} } $$

where we took into consideration that the magnetic field is orthogonal to the current that generated it. Substitution of Eqs. (19a) and (19b) into Eqs. of (8a) and (8b), respectively, yields

20a $$ R_{1} = \frac{{\sqrt[4]{{\pi f\rho_{1} \mu_{2} }}\sqrt[4]{{\pi f\rho_{2} \mu_{1} }}}}{2\pi }\ln \left( {1 + \frac{{\sqrt {\pi f\rho_{1} \mu_{2} } }}{{\sqrt {\pi f\rho_{2} \mu_{1} } }}} \right) $$

and

20b $$ R_{2} = \frac{{\sqrt[4]{{\pi f\rho_{1} \mu_{2} }}\sqrt[4]{{\pi f\rho_{2} \mu_{1} }}}}{2\pi }\ln \left( {1 + \frac{{\sqrt {\pi f\rho_{2} \mu_{1} } }}{{\sqrt {\pi f\rho_{1} \mu_{2} } }}} \right) $$

In the case of magnetic anisotropy only ($\rho_{1} = \rho_{1} = \rho$), the above equations simplify to

21a $$ R_{1} = \frac{{\sqrt {\pi f\rho } \sqrt[4]{{\mu_{1} \mu_{2} }}}}{2\pi }\ln \left( {1 + \frac{{\sqrt {\mu_{2} } }}{{\sqrt {\mu_{1} } }}} \right) $$

and

21b $$ R_{2} = \frac{{\sqrt {\pi f\rho } \sqrt[4]{{\mu_{1} \mu_{2} }}}}{2\pi }\ln \left( {1 + \frac{{\sqrt {\mu_{1} } }}{{\sqrt {\mu_{2} } }}} \right) $$

In the absence of stress-induced magnetic anisotropy ($\mu_{1} = \mu_{2} = \mu$)

22 $$ R_{10} = R_{20} = \frac{{\sqrt {\pi f\rho \mu } }}{2\pi }\ln \left( 2 \right) $$

Then, the normalized resistances can be obtained as follows

23a $$ \frac{{R_{1} }}{{R_{10} }} = \frac{{\sqrt[4]{{\mu_{1} \mu_{2} }}}}{\sqrt \mu }\frac{{\ln \left( {1 + \sqrt {\frac{{\mu_{2} }}{{\mu_{1} }}} } \right)}}{\ln \left( 2 \right)} $$

and

23b $$ \frac{{R_{2} }}{{R_{20} }} = \frac{{\sqrt[4]{{\mu_{1} \mu_{2} }}}}{\sqrt \mu }\frac{{\ln \left( {1 + \sqrt {\frac{{\mu_{1} }}{{\mu_{2} }}} } \right)}}{\ln \left( 2 \right)} $$

Finally, the normalized resistance ratio measured on the anisotropic material can be obtained as

24 $$ \frac{{R_{1} }}{{R_{10} }}/ \frac{{R_{2} }}{{R_{20} }} = \frac{{\ln \left( {1 + \sqrt {\frac{{\mu_{2} }}{{\mu_{1} }}} } \right)}}{{\ln \left( {1 + \sqrt {\frac{{\mu_{1} }}{{\mu_{2} }}} } \right)}} $$

**Fig. 6:**
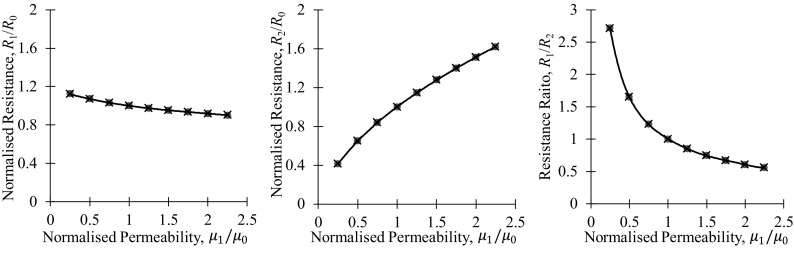
Comparison of simulated results (red circles) and the approximations of Eq. (23) and (24) (black lines and crosses) for a square electrode configuration AC potential drop measurement in a magnetically anisotropic thick plate. The permeability in the $\varvec{x}_{1}$ direction was varied from 25 to 225 while the other components were kept at 100 (Color figure online)

These results have been validated using a finite element model of a square electrode configuration on a thick magnetically anisotropic cuboid of thickness 50 mm and electrode separation 10 mm, the results are shown in Fig. 6.

This analysis illustrates that the potential drop measurements are primarily sensitive to permeability changes normal to the direction of current injection; the magnetic field is generated in the plane normal to the current flux and so will be sensitive to the current field in that direction.

Although less relevant to the present study, it is worth pointing out that following the same logic as Eqs. (21a, 21b, 22, 23a, 23b, 24), an equivalent equation for cases of electrical anisotropy can be found [2],

25a $$ \frac{{R_{1} }}{{R_{10} }} = \frac{{\sqrt[4]{{\rho_{1} \rho_{2} }}}}{\sqrt \rho }\frac{{\ln \left( {1 + \sqrt {\frac{{\rho_{1} }}{{\rho_{2} }}} } \right)}}{\ln \left( 2 \right)} $$

and

25b $$ \frac{{R_{2} }}{{R_{20} }} = \frac{{\sqrt[4]{{\rho_{1} \rho_{2} }}}}{\sqrt \rho }\frac{{\ln \left( {1 + \sqrt {\frac{{\rho_{2} }}{{\rho_{1} }}} } \right)}}{\ln \left( 2 \right)} $$

With the normalised resistance ratio being,

26 $$ \frac{{R_{1} }}{{R_{10} }}/ \frac{{R_{2} }}{{R_{20} }} = \frac{{\ln \left( {1 + \sqrt {\frac{{\rho_{1} }}{{\rho_{2} }}} } \right)}}{{\ln \left( {1 + \sqrt {\frac{{\rho_{2} }}{{\rho_{1} }}} } \right)}} $$

**Fig. 7:**
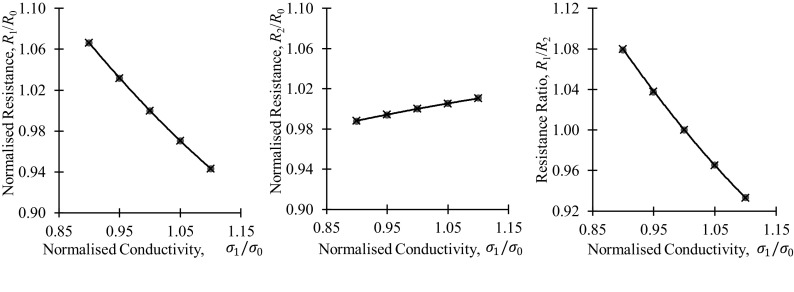
Comparison of simulated results (red circles) and the approximations of Eq. (25) and (26) (black lines and crosses) for a square electrode configuration AC potential drop measurement in an electrically anisotropic thick plate

Again, these results have been validated using a finite element model of a square electrode configuration on a thick magnetically anisotropic cuboid of thickness 50 mm and electrode separation 10 mm, the results are shown in Fig. 7. Unlike permeability, the potential drop measurements are primarily sensitive to resistivity changes parallel to the direction of current injection, the net direction of current flow.

The excellent fit between the simulated data and analytical approximations on these figures proves the high accuracy of the ± 1/4 power regression lines previously indicated on Figs. 4e and f.

## Influence of Elastic Strain on Directional PD Measurements

In this section an experimental demonstration will be conducted to show that AC resistance measurements may be used as an indicative metric of applied stress. From the previous work of Dover et al. [7, 8, 14, 30, 31] uniaxial loading is expected to cause an anisotropic change in magnetic permeability. In tension the permeability is expected to increase in the direction parallel to loading at the expense of the remaining two; in the case of uniaxial loading the elastic strain and therefore change in permeability in the two directions normal to loading are expected to be equal. For small changes in strain this may be assumed to be linear so that, assuming loading is in the $x_{2}$ direction,

27 $$ \mu = \left( {\begin{array}{*{20}c} {\mu_{0} \mu_{r} - \beta \varepsilon \mu_{0} \left( {\mu_{r} - 1} \right)} & 0 & 0 \\ 0 & {\mu_{0} \mu_{r} + \alpha \varepsilon \mu_{0} \left( {\mu_{r} + 1} \right)} & 0 \\ 0 & 0 & {\mu_{0} \mu_{r} - \beta \varepsilon \mu_{0} \left( {\mu_{r} - 1} \right)} \\ \end{array} } \right) $$

where $\varepsilon$ is the elastic strain in the loading direction and $\alpha$ and $\beta$ are constants relating the change in strain to permeability. The assumption that the stress-dependent change in permeability is linear is expected to be limited as the change in permeability is expected to become increasingly saturated as the number of magnetic domains available for reorientation diminishes.

The subsequent influence of permeability on AC potential drop measurements can then be inferred from Eqs. (23) and (24). An exact inversion between AC potential drop measurements and stress, via magnetic permeability would therefore at least require the parameters $\alpha$ and $\beta$ which will certainly be material specific and may also be dependent on many different variables, as will be explored in the following section. It will also be required that the saturating effect be minimal.

**Fig. 8:**
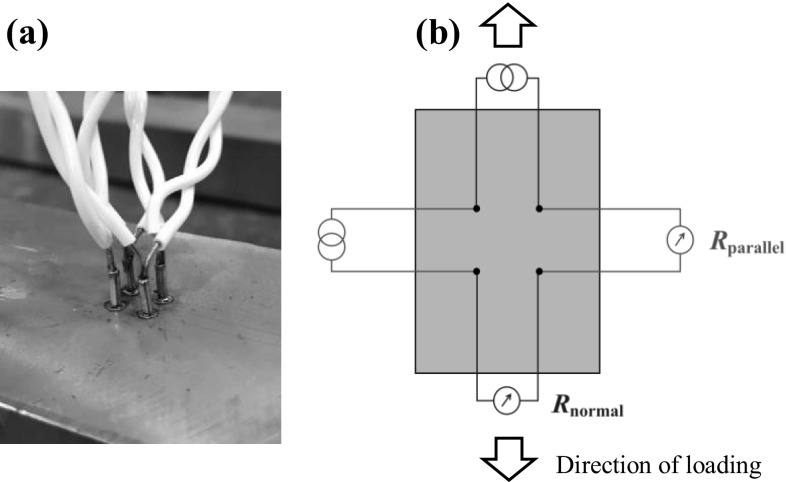
a Photograph showing the square arrangement of electrodes, for scale the electrode separation is 5 mm. **b** Illustration showing the ‘parallel’ and ‘normal’ notation used in this section

To illustrate the stress dependence of AC potential drop measurements through magnetic permeability two experiments were conducted; a ferritic S275 structural steel and a less permeable F51 duplex steel. Bars of material 20 mm × 50 mm × 750 mm were prepared with a square configuration of electrodes with 5 mm electrode separation; the electrodes were formed by 1.5 mm diameter stainless steel studs spot welded onto the component, as shown in Fig. 8a. For the remainder of the paper the notation will be used that ‘parallel’ measurements are those where the injection and sensing electrodes are aligned in the long axis of the component while in ‘normal’ measurements the injection and sensing electrodes are aligned in the short axis of the component, as shown in Fig. 8b. Prior to testing, the components were demagnetised by passing them through a Magnaflux L10 coil [22] with 13A mains frequency current. The applied load was increased from 0 to 100 MPa in 20 MPa steps, then from 100 MPa down to − 100 MPa and back up to 0. The cycling was repeated 10 times.

**Fig. 9:**
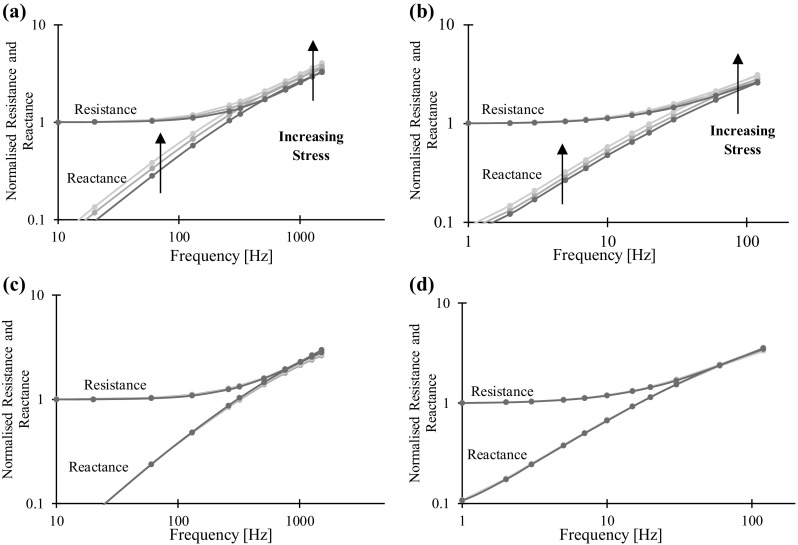
Example frequency responses of square configuration 5 mm electrode separation potential drop measurements. Resistance and Reactance normalised to the 1 Hz resistance. Top row: measurements normal to loading direction. Bottom row: measurements parallel to loading direction. Left column: duplex steel. Right column: ferritic steel. Loads of -100 MPa (blue), 0 MPa (green) and 100 MPa (yellow) shown (Color figure online)

Impedance measurements were taken using a lock-in amplifier based system. A typical set of impedance-frequency measurements are shown in Fig. 9 for both materials and a range of stresses. The frequency response closely reflects that predicted by theory in Fig. 3. The ferritic steel has a much higher magnetic permeability than the duplex steel so that the transition between DC and AC behaviour occurs at a much lower frequency. Changes in the elastic strain alters the magnetic permeability and therefore the skin effect. The DC-asymptote of the resistance is not affected by the skin effect and therefore is independent of the applied stress. Because of the inevitable magnetic coupling between the injection and sensing loops, PD probes exhibit a small inherent mutual inductance that results in a measured reactance that is linearly proportional to frequency but still much smaller than the corresponding resistance in the quasi-DC range. The AC behaviour is dictated by the skin effect and therefore is sensitive to applied stress. It is clear that the perpendicular measurements are much more responsive to the applied load; from Eqs. (23a, 23b) this is consistent with a greater change in permeability in the loading direction, which is expected due to the larger strain. To reduce the number of measurements, only 1 and 1510 Hz measurements were collected for the duplex steel and 1 and 120 Hz for the ferritic steel for the remaining experiments. We have used the term quasi-DC throughout although it is implied by the 1 Hz frequency as we feel it is insightful (a 1 Hz frequency in many cases is not quasi-DC). Only the resistance will be displayed for brevity; the same stress dependence can clearly be derived from the reactance at all frequencies although it is worth noting that at low frequencies reactance is small and therefore more difficult to measure.

**Fig. 10:**
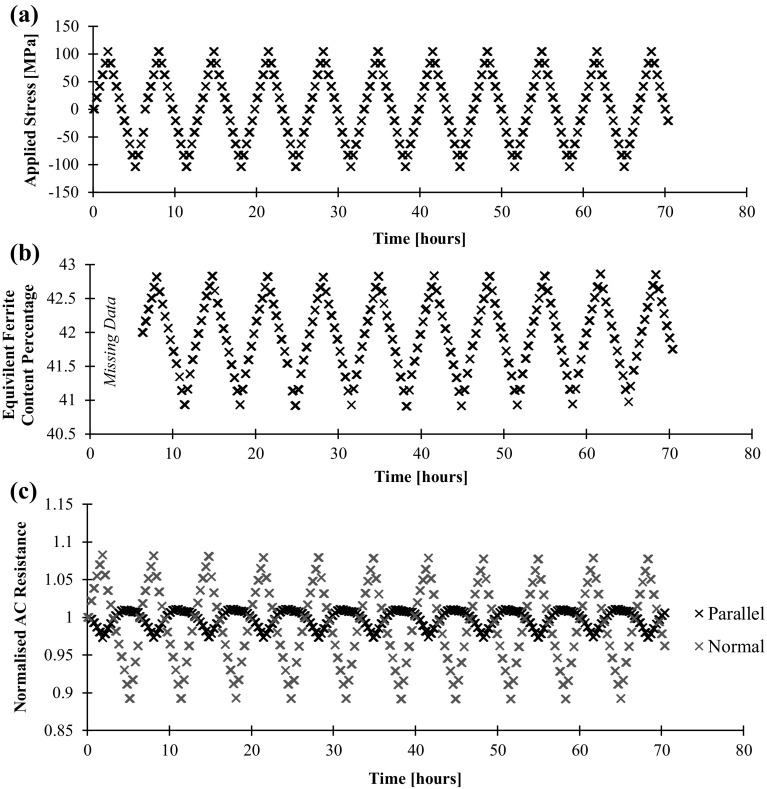
a Applied stress, **b** Feritscope reading as equivalent ferrite content percentage, **c** AC potential drop resistance measurements against time for the duplex steel test component. In this case the AC frequency is 1510 Hz

A Fischer FMP30 Feritscope [13] probe was also fixed to the component in order to obtain an independent, albeit, essentially non-directional metric of permeability. The instrument equates permeability to ferrite content and therefore displays readings as percentage equivalent ferrite content (%EFC). Magnetic permeability and %EFC may be approximately converted using the empirical data presented by Yin et al. [29]; following this approach, as a rough estimate, the duplex steel and mild steel have a relative magnetic permeability of approximately 40 and 200 respectively. Figure 10 shows how both the Feritscope reading and AC resistance change over a number of stress cycles for the duplex steel. Again, it is clear that the measurements made in the normal direction are much more affected by the stress cycles. It is additionally clear that the parallel measurements appear to saturate at large tensile strains. Comparing the potential drop measurements and the non-directional Feritscope measurements is informative; when the component is in tension the permeability in the loading direction increases more than it decreases in the normal direction, this is reflected by a larger change in the normal potential drop measurement than the parallel reading and also a net decrease in the Feritscope reading.

**Fig. 11:**
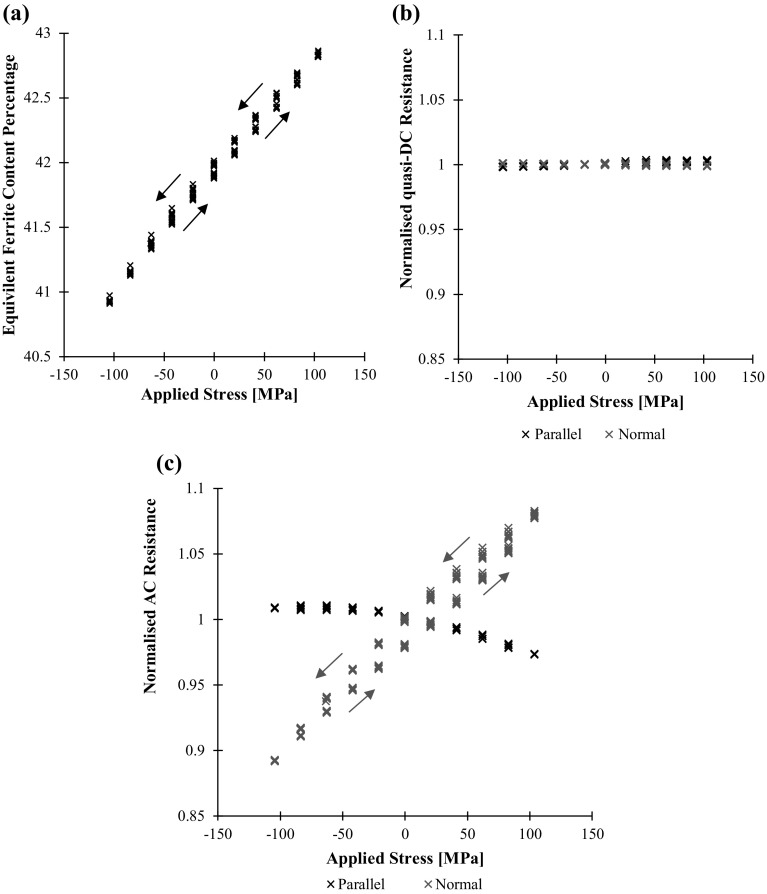
a Feritscope reading as equivalent ferrite content percentage, **b** quasi-DC potential drop measurement, **c** AC potential drop measurement against applied stress for the duplex steel test component. Results over the 11 stress cycles of Fig. 10 shown. In this case the quasi-DC frequency is 1 Hz and the AC frequency is 1510 Hz

The same data, plus additionally the quasi-DC resistance measurements, are displayed as measurement against applied stress in Fig. 11. The permeability and therefore stress dependence of both the Feritscope and the AC resistance readings is very clear and can be measured with good accuracy over this range. This figure shows the repeatability of the measurements over subsequent cycles, but also shows hysteresis that afflicts many magnetic based techniques; as the magnetic domains become ordered by the imposition of strain they remain ordered until the strain is reversed. The hysteresis provides a major obstacle in inverting readings to strain, unless measurements are made continuously so that the hysteresis is tracked. It is worth of noting that there is no difference between the initial and subsequent cycles, i.e., the weak stress-induced magnetisation follows a stable “minor loop” without irreversible change of the magnetic domains in the sample under test.

It is important to note that the observed changes in resistance are not a consequence of the geometric influence of electrode separation [10] or piezoresistivity as evident by the negligible changes in quasi-DC resistance shown in Fig. 11b.

**Fig. 12:**
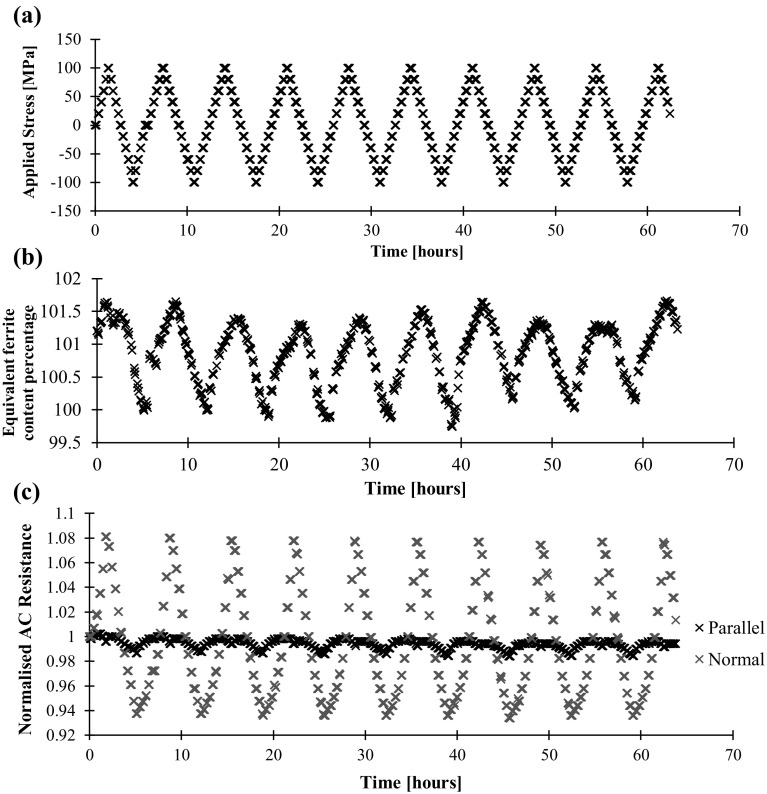
a Applied stress, **b** Feritscope reading as equivalent ferrite content percentage, **c** AC potential drop resistance measurements against time for the ferritic steel test component. In this case the AC frequency is 1510 Hz

The ferritic steel was then tested in the same way. The Feritscope and AC resistance measurements are shown in Fig. 12b, c respectively. Clearly, the Feritscope measurements are less reproducible from cycle to cycle. It is hypothesised that this is due to the nonlinear measurement characteristics of the instrument; it is at the upper limit of the instrument design range where the sensitivity to changes in magnetic permeability decreases. Again, as anticipated, the normal AC potential drop measurements are much more affected by the applied load than the parallel; in this case both directions show signs of saturation.

**Fig. 13:**
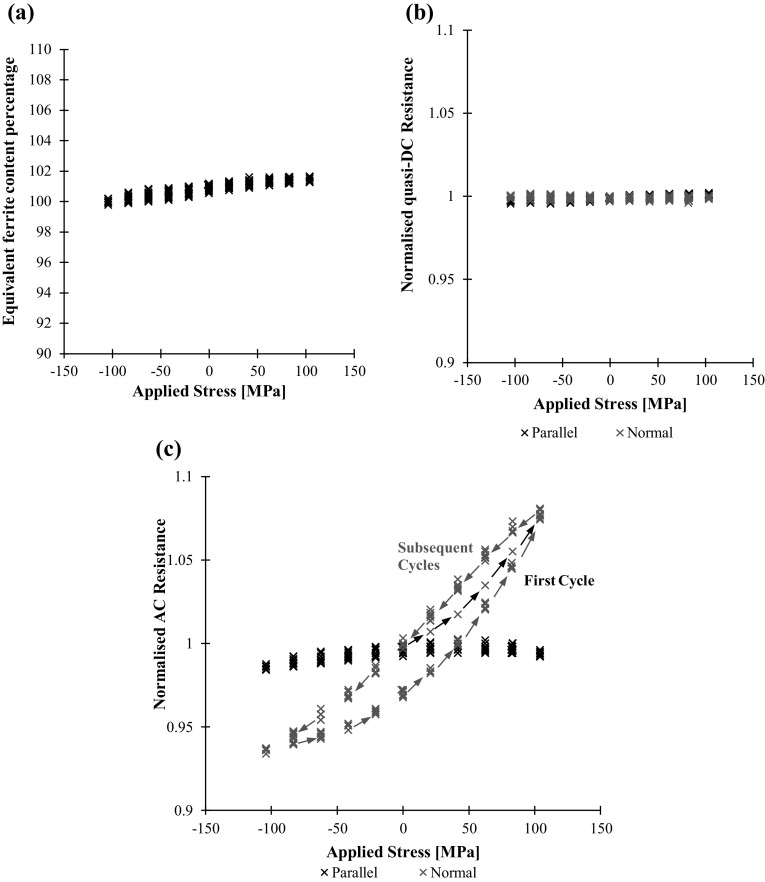
a Feritscope reading in equivalent ferrite content percentage, **b** quasi-DC potential drop measurement, **c** AC potential drop measurement against applied stress for the ferritic steel test component. Results over the 10 stress cycles of this figure shown. In this case the quasi-DC frequency is 1 Hz and the AC frequency is 120 Hz

Figure 13 shows the Feritscope and potential drop measurements against applied stress. In the ferritic mild steel material with much higher permeability the Feritscope readings have a much weaker correlation with applied stress. The AC resistance readings however have a very clear and repeatable stress dependence. The hysteresis is again present, though this time the distinct behaviour of the initial cycle is also evident. For completeness, the DC resistance measurements are included to demonstrate that the observed AC response is not a consequence of gross geometry change or piezoresistivity.

## Specificity: Influence of Temperature and Magnetisation

The potential drop technique is evidently sensitive to applied stress and the changes are clearly measureable. The primary issue in whether magnetic permeability can be exploited for practical engineering applications is selectivity: whether additional variables also influence readings, undermining a possible stress inversion.

The proposed potential drop technique relies on the dependence of the skin effect on stress-dependent permeability. From Eq. (1) it is evident that both electrical resistivity and magnetic permeability determine the skin effect and so changes in either could be problematic. In principle, assuming there is no gross change is geometry then resistivity may be monitored using the permeability independent quasi-DC resistance and therefore compensated against; we will therefore focus primarily on permeability.

As noted in the introduction, numerous intrinsic parameters influence the magnetic permeability: alloy content, microstructural state and cold work form part of a non-exhaustive list that may cause long term ‘drift’ in permeability. Two parameters that are known to influence magnetic permeability are temperature and external magnetising field [17]. In order to explore this limitation further the temperature and magnetisation of the test component will be manipulated while measurements are taken. The duplex material will be used for this study due to the superior Feritscope measurements.

### Temperature

The same duplex material bar used in the stress experiments was demagnetised and placed in an environmental chamber, together with the Feritscope probe and the potential drop electrodes, all other measurement equipment remained outside of the chamber. The temperature was cycled between 10 and 50 °C 3 times with a period of 40 h.

**Fig. 14:**
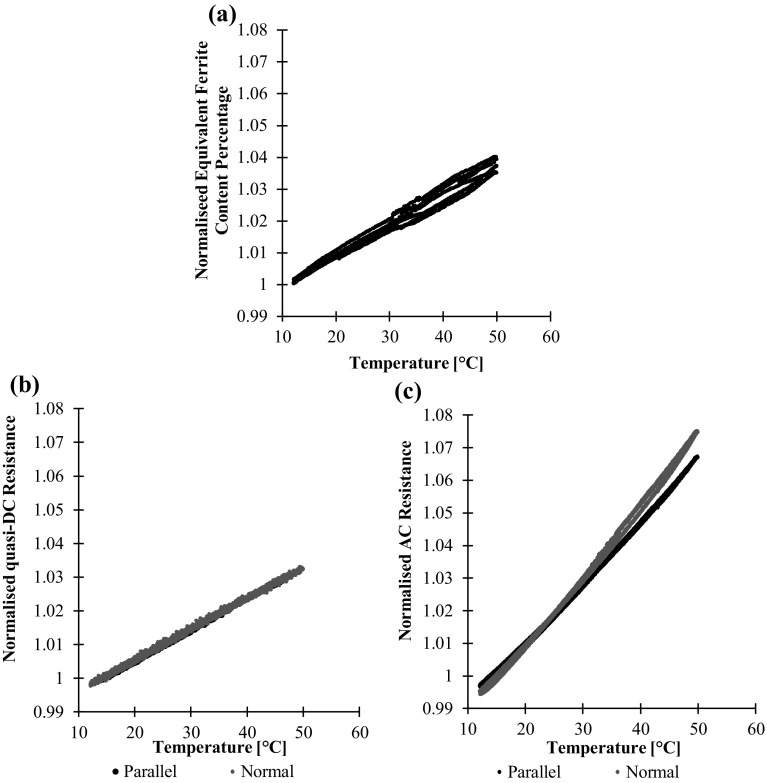
a Feritscope reading, **b** quasi-DC potential drop resistance and **c** AC potential drop resistance measurements against duplex steel test component temperature. In this case the quasi-DC frequency is 1 Hz and the AC frequency is 1510 Hz

Figure 14 shows the Feritscope, DC and AC potential drop measurements against temperature. The DC measurements show the temperature dependence of electrical resistivity; the resistivity changes are extremely repeatable and isotropic as anticipated. The non-directional Feritscope measurements show a strong albeit not particularly repeatable correlation with temperature. The AC potential drop measurements, sensitive to both directional electrical resistivity and permeability changes are seen to be strongly influenced; this is anticipated due to the dual sensitivity to both resistivity and permeability, both of which are exceptionally temperature sensitive [4, 23]. The changes are very repeatable, although not perfectly isotropic; this must be a consequence of directional changes in permeability with temperature, perhaps due to the long slender geometry. While the influence shown over this modest temperature range are reasonably linear, we must be mindful that this is not the case over a wider temperature range, particularly approaching the Curie temperature where we expect strong and non-monotonic changes in permeability [4].

The magnitude of the changes in AC resistance due to the 40 °C change is similar to that resulting from a 100 MPa imposed stress. Whether the temperature sensitivity will be problematic will depend on the anticipated range of applied stress and the temperature for that particular situation, but clearly the influence of temperature must be carefully considered. It is likely that the temperature dependence may be compensated by a variety of strategies; the simplest is using a direct temperature measurement, or indeed the resistivity dependent DC resistance measurement, and using simple regression. As the temperature dependence of magnetic permeability is apparently anisotropic, each direction will need to be compensated for individually and the compensation parameters may need to be regularly revised. Likewise, if the loading was known a priori to be uniaxial, then it may be possible to separate the influence of isotropic changes from the strongly anisotropic influence of stress, though again the effectiveness will be limited by the apparently anisotropic temperature dependence.

### Magnetisation

**Fig. 15:**
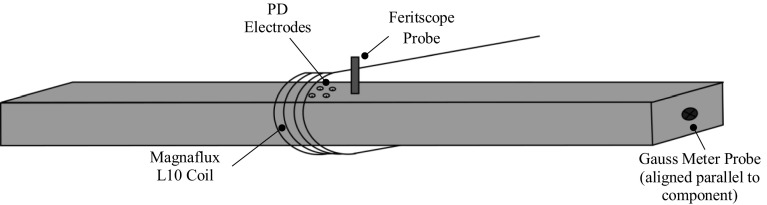
Illustration of the magnetisation experiment

The general arrangement of the magnetisation experiment is shown in Fig. 15. The duplex test specimen was placed inside a ~ 3000-turn Magnaflux L10 coil powered with up to ± 3A direct current in order to impose a magnetic field. The Feritscope and potential drop electrodes remained unchanged in the centre of the component, in the vicinity of the coil. A Hirst GM08 Gaussmeter [16] was taped to a fixed position approximately in the centre of the end of the component in order to get an indication of the magnetisation; note that this provides a quantitative measure of the magnetisation of the component as opposed to that created by the coil.

**Fig. 16:**
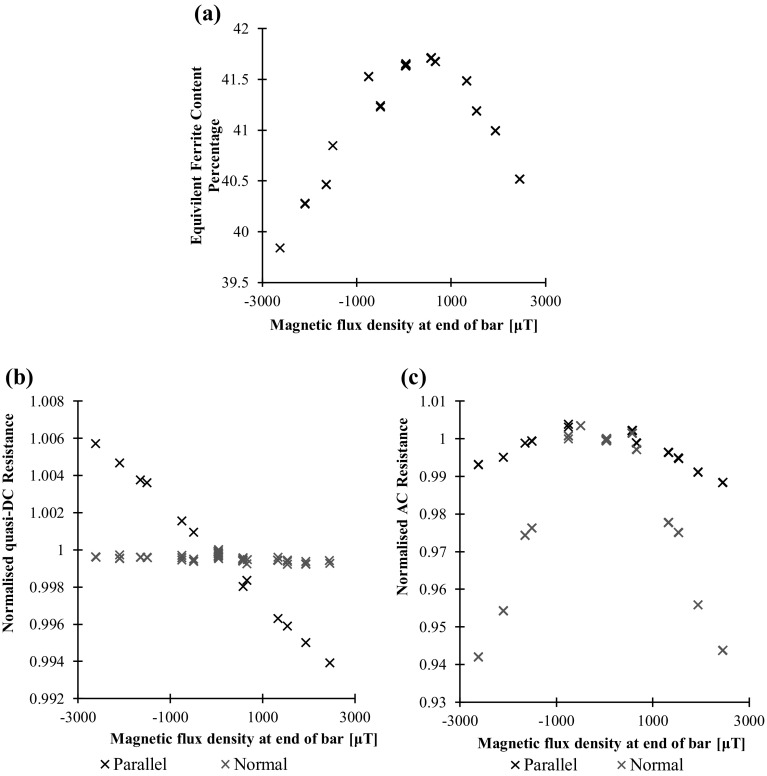
a Feritscope reading in equivalent ferrite content percentage, **b** quasi-DC potential drop resistance and **c** AC potential drop resistance measurements against Gauss meter reading. In this case the quasi-DC frequency is 1 Hz and the AC frequency is 1510 Hz

Figure 16 shows the Feritscope and the DC and AC potential drop measurements against magnetic flux density at the end of the bar. It should be noted that the magnetisation obtained in this experiment is orders of magnitude larger than what might be expected from the geomagnetic field in order to produce clearly measureable trends, for context the Earth’s magnetic field is of the order of 30 μT. From the Feritscope and AC potential drop measurements the greater the magnetic field the lower the permeability; this may be explained as the magnetic field orders the magnetic domains reducing the magnetic domains available for reversible reorientation by the AC magnetic field of the injected current. The behaviour is similar regardless of the polarity of the magnetisation as the same saturating effect occurs in both directions. Again, the directional AC potential drop measurements reveal that the permeability change is much greater in the direction of magnetisation, therefore influencing the normal measurement much more. It may be concluded that below < 500 μT, the range of practical interest, the influence on the AC measurements is < 1%, again, the significance of this depends on the anticipated stress range.

As an aside, it is interesting to observe the magnetisation dependence of the quasi-DC measurements. The behaviour is quite different from all the other measurements: there is negligible change in the direction normal to the magnetisation and an approximately linear relationship in the parallel direction. This relatively weak (≪ 1%), although clearly measurable effect, is anisotropic magnetoresistance [4, 25].

## Discussion

The ability of the proposed potential drop technique to monitor stress-dependent, anisotropic, changes in magnetic permeability has been demonstrated; large, directional changes in impedance result from the imposition of stress. The two orthogonal impedance measurements are sensitive to the two components of the magnetic permeability tensor tangential to the surface. These two magnetic permeability components are determined by the three orthogonal elastic strain components, and therefore inversion to find strain is possible only if Poisson’s ratio is a priori known. For elastic solids Poisson’s ratio is one of the more stable material properties that can be usually estimated within ± 0.02 or better than ± 10%. Compared to intrinsic uncertainties of the magnetic properties, that is effectively a known constant. As an example, in the present paper a uniaxial stress is imposed, and so the three strains are related by Poisson’s ratio only (if uniaxial stress $\sigma_{1}$ is imposed in the $x_{1}$ direction $\varepsilon_{2} = \varepsilon_{3} = - v\varepsilon_{1}$), then an inversion similar to Eq. (27) may be used. In the more general case of biaxial tangential stress ($\sigma_{1} ,\sigma_{2}$) the three strains are again related by Poisson’s ratio, only $\varepsilon_{3} = - \left( {\varepsilon_{3} + \varepsilon_{3} } \right)v/\left( {1 - v} \right)$, so we get a slightly modified version of Eq. (27), but inversion is still possible. Nonetheless, the potential drop technique provides a very clear indication of imposed stress, which may for example be of use in measuring relative size and number of stress cycles for fatigue analysis.

Magnetic hysteresis is a significant impediment in the interpretation of isolated permeability measurements as solutions will not be unique. In previous studies hysteresis was suppressed by demagnetisation [7], though this is not an approach that is suitable for practical engineering situations. Continuous measurements, where practical, would enable the tracking of hysteresis and therefore may permit interpretation.

The accuracy of the proposed magnetic stress monitoring technique using directional potential drop measurements is limited by the highly uncertain relationship between elastic stress and magnetic permeability, which depends on a number of material parameters as well as on magnetic history. This is an inherent limitation of all magnetic stress measurements in ferromagnetic materials and can be controlled only by adopting empirical calibration procedures. Despite the resistance measurement repeatability of typically better than 0.1% during static conditions, we can observe an approximately ± 10% variation in AC resistance that corresponds to about ± 10 MPa or ± 10% stress uncertainty (see for example the ± 100 MPa applied stress measurement of Fig. 11).

Frequently, lack of specificity undermines magnetic measurements. In this study the influence of temperature and magnetisation were investigated. The strong temperature dependence of both resistivity and permeability has been shown to cause large changes in both DC and AC resistance. Whether the effect of temperature dependence can be neglected depends on the anticipated stress range and temperature range. In this investigation, 40 °C temperature change has a similar magnitude effect as 100 MPa applied stress, which is expected to be approximately half the yield strength. It is possible that in very temperature stable situations (e.g. biomedical or sub-sea) and where stresses are large then the influence of temperature will be acceptably small, but otherwise some attempt should be made to compensate. Importantly, the influence of uniaxial stress is directional, while temperature is much more (though not perfectly) isotropic. This difference enables a possibility in separating the two effects, though the slight anisotropy in the temperature dependence provides a challenge in reliable temperature compensation. The influence of temperature on the stress sensitivity has not been explored in this study. As an extreme example, approaching the Curie temperature the ordering effect of elastic strain would have to compete against the disordering effect of temperature, which is likely to lower the strain sensitivity. Whether this is significant over a modest temperature range should be investigated in future research.

Magnetisation may also pose a problem, particularly in long slender components that may be susceptible to spontaneous macroscopic magnetisation. It is important to emphasise the magnetic field used in this study is many orders of magnitude larger than would be encountered in a typical engineering situation; provided there is nothing of note to drive the magnetisation, it is unlikely to be significantly problematic. The effect of magnetisation on the stress sensitivity should be explored in future.

Various ‘intrinsic’ material parameters will also influence the magnetic permeability. It is possible that microstructural evolution will additionally influence the magnetic permeability, again adding complexity to a possible inversion. Such changes are likely to be long-term, monotonic, and approximately isotropic and may therefore be separable from the influence of at least dynamic loading. It has been suggested in previous literature that it may be possible to use magnetic properties to monitor the microstructural state of engineering components [18–20]. This study has shown that various parameters including stress state, temperature and magnetisation cause significant changes in magnetic permeability and are likely to dominate the subtle influence of microstructural evolution.

It is important to remember that the information on the permeability-dependent skin effect may be obtained as a by-product of an existing potential drop measurement. Potential drop measurements are usually deployed for monitoring geometry changes associated with a particular damage mode such as crack size or strain. In ferromagnetic materials DC or quasi-DC is used to suppress the influence of the skin effect, it is proposed that the same hardware may be used to additionally take an AC measurement which may reveal additional useful information on stress state. It is also possible to extract the same information from the quasi-DC reactance without taking any additional measurements, albeit the quasi-DC reactance will be small and difficult to measure reliably because of inevitable inductive cross-talk between the injecting and sensing pair of cables. A suitable use might therefore be the monitoring of the number and relative size of fatigue stress cycles where the potential drop sensor may also be used to monitor crack size.

## Conclusions

A potential drop technique has been presented that is capable of measuring the anisotropic changes in permeability resulting from the inverse magnetostrictive effect. An ad hoc approximation has been developed which captures the main features of the sensitivity of the technique to anisotopic changes in resistivity and magnetic permeability.

The sensitivity to applied stress has been demonstrated experimentally in uniaxial loading. The behaviour is broadly consistent with what is anticipated from the ad hoc description, yet there are complicating features of magnetism that are not included in the simplified analysis, which limit the ability to accurately invert measurements; saturation and hysteresis are evident in measurements which will need to be accounted for.

Selectivity is frequently an issue in magnetic measurements, it has been shown that aside from the influence of elastic strain, temperature and magnetisation also influence permeability and therefore may undermine a stress inversion. Additionally, long-term material evolution may also influence measurements.

With the above in mind, applications where stress monitoring using the proposed potential drop technique is appropriate must be carefully selected. Particularly suitable applications are those where:

- The monitored component is ferromagnetic
- The temperature is relatively stable or compensated against, and there are no exceptionally strong static magnetic fields present
- The stresses of interest are non-stationary, so that they may be separated from spurious long-term ‘drift’ in permeability
- The multiaxiality of the stress state is known or is at least repeatable
- The situation is not suitable for conventional strain gauges

It is proposed that a broad opportunity where potential drop stress measurements may be suitable is monitoring the number and relative size of fatigue cycles. This may be particularly attractive as the stress information may be obtained as a by-product of an existing potential drop measurement for crack sizing.
